# Multivalvular Endocarditis: A Rare Condition with Poor Prognosis

**DOI:** 10.3390/jcm11164736

**Published:** 2022-08-13

**Authors:** Sara Álvarez-Zaballos, Victor González-Ramallo, Eduard Quintana, Patricia Muñoz, Sofía de la Villa-Martínez, M. Carmen Fariñas, Francisco Arnáiz-de las Revillas, Arístides de Alarcón, M. Ángeles Rodríguez-Esteban, José M. Miró, Miguel Angel Goenaga, Josune Goikoetxea-Agirre, Elisa García-Vázquez, Lucía Boix-Palop, Manuel Martínez-Sellés, on behalf of GAMES

**Affiliations:** 1 Cardiology Department, Hospital General Universitario Gregorio Marañón, 28027 Madrid, Spain; 2 Home Hospitalization Department, Hospital General Universitario Gregorio Marañón, 28027 Madrid, Spain; 3 Cardiovascular Surgery Department, Hospital Clinic, 08036 Barcelona, Spain; 4 Clinical Microbiology and Infectious Diseases Department, Hospital General Universitario Gregorio Marañón, Health Research Institute Gregorio Marañón, 28027 Madrid, Spain; 5 CIBER Enfermedades Respiratorias-CIBERES (CB06/06/0058), School of Medicine, Universidad Complutense de Madrid, 28040 Madrid, Spain; 6 Infectious Diseases Department, Hospital Universitario Marqués de Valdecilla, IDIVAL, Universidad de Cantabria, 39008 Santander, Spain; 7 CIBER de Enfermedades Infecciosas-CIBERINFEC (CB21/13/00068), Instituto de Salud Carlos III, 28029 Madrid, Spain; 8 Clinical Unit of Infectious Diseases and Clinical Microbiology, Infectious Diseases Research Group, Institute of Biomedicine of Seville (IBIS), University of Seville/CSIC/University Virgen del Rocío, 41013 Seville, Spain; 9 Cardiovascular Surgery ICU Department, Hospital Central de Asturias, 33011 Oviedo, Spain; 10 Infectious Diseases Service, Hospital Clínic—IDIBAPS, University of Barcelona, 08036 Barcelona, Spain; 11 Infectious Diseases Department, Hospital Universitario Donosti, 20014 San Sebastián, Spain; 12 Infectious Diseases Department, Hospital Universitario de Cruces, 48903 Bilbao, Spain; 13 Internal Medicine-Infectious Diseases Department, IMIB, Hospital Clínico Universitario Virgen de la Arrixaca, Facultad de Medicina, Universidad de Murcia, 30120 Murcia, Spain; 14 Infectious Diseases Department, Hospital Universitari Mútua Terrassa, 08221 Barcelona, Spain; 15 CIBERCV (CIBER Enfermedades Cardiovasculares), Universidad Europea, Universidad Complutense, 28040 Madrid, Spain

**Keywords:** infective endocarditis, multivalvular endocarditis, prognosis, mortality

## Abstract

Background. Infective endocarditis (IE) is a severe condition. Our aim was to describe the profile and prognosis of patients with multivalvular infective endocarditis (MIE) and compare them to single-valve IE (SIE). Methods. We used a retrospective analysis of the Spanish IE Registry (2008–2020). Results. From 4064 definite cases of valvular IE, 577 (14.2%) had MIE. In patients with MIE, the most common locations were mitral (552, 95.7%) and aortic (550, 95.3%), with mitral-aortic involvement present in 507 patients (87.9%). The most common etiologies were *S. viridans* (192, 33.3%) and *S. aureus* (113, 19.6%). MIE involved only native valves in 450 patients (78.0%). Compared with patients with SIE, patients with MIE had a similar age (69 vs. 67 years, respectively, *p* = 0.27) and similar baseline characteristics, but were more frequently men (67.1% vs. 72.9%, *p* = 0.005) and had a higher incidence of intracardiac complications (36.2% vs. 50.4%, *p* < 0.001), heart failure (42.7% vs. 52.9%, *p* < 0.001), surgical indication (67.7 vs. 85.1%, *p* < 0.001), surgery (46.3% vs. 56.3%), and in-hospital mortality (26.9% vs. 34.3%, *p* < 0.001). MIE was an independent predictor of in-hospital mortality (odds ratio (OR) 1.3, 95% confidence interval (CI) 1.1–1.7, *p* = 0.004) but did not have an independent association with 1-year mortality (OR 1.1, 95% CI 0.9–1.4, *p* = 0.43). Conclusions. About one-seventh of the valvular IE patients had MIE, mainly due to mitral-aortic involvement. MIE is associated with a poor in-hospital prognosis. An early diagnosis and treatment of IE might avoid its spread to a second valve.

## 1. Introduction

Infective endocarditis (IE) is a severe condition associated with high mortality and frequent complications [1]. Multivalvular IE (MIE) is relatively uncommon, with incidence ranging from 12% to 30% [2,3]. MIE information is scarce, and most published data come from case reports and surgical treatment techniques [3,4,5,6,7,8,9,10]. Few studies have compared MIE with single-valve IE (SIE) [2,9,11]. The prognostic influence of MIE is unclear as some data suggest an association with poor outcomes [3,4,5,12,13,14,15], whereas others do not [2,6,7,8,9,11]. The aim of our study was to describe the profile and prognosis of patients with MIE and to compare them with SIE in a large cohort of IE patients.

## 2. Materials and Methods

The Spanish Collaboration on Endocarditis, *Grupo de Apoyo al Manejo de la endocarditis infecciosa en ESpaña* (GAMES), is a national observational prospective registry that has been previously described [16,17,18,19]. Multidisciplinary teams, including infectious disease physicians, cardiologists, cardiac surgeons, microbiologists, echocardiographers, and other imaging specialists, have completed standardized case report forms with information regarding IE episodes and follow-up data. A complete list of GAMES members is shown in Appendix A. IE patients at 38 Spanish hospitals between January 2008 and 2020 were included. Inclusion criteria were the diagnosis of definite valvular IE by modified Duke criteria [20]. IE management, including the decision to perform surgery and the type of surgery, was performed by the local medical team following the 2009 and 2015 European Society of Cardiology recommendations [1]. MIE was considered present when two or more valves were involved. Valve involvement was defined by echocardiography as valves with vegetations or new regurgitation or by direct intraoperative visualization of vegetations.

This study complied with the principles outlined in the Declaration of Helsinki and was approved by the ethics committee of participating centers.

### Statistical Methods

Continuous variables are summarized as means ± standard deviations (SDs) or medians and interquartile ranges when normal distribution was not observed, as per the Kolmogorov–Smirnov goodness-of-fit test; categorical variables are expressed as numbers and percentages. Student‘s *t*-test, Mann–Whitney U test, or paired *t*-test was used to compare the continuous variables. The categorical variables were compared using the χ^2^ test or Fisher’s exact test. Kaplan–Meier curves were used to assess the cumulative survival of patients with valvular IE according to the presence of SIE or MIE. The curves were compared with the log-rank test. Multivariable logistic regression analyses (backward selection) were performed to determine the mortality predictors and to assess the independent association of MIE with mortality. All variables with a *p*-value < 0.10 in univariate analyses were included in the multivariable analyses. The statistical analysis was performed using SPSS, version 22.0 (IBM, Armonk, NY, USA).

## 3. Results

**Figure 1:**
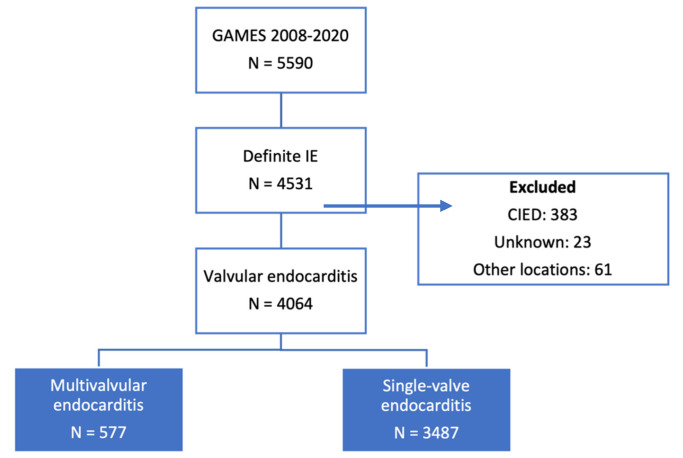
Flow chart of our cohort of patients from the Spanish Collaboration on Endocarditis, *Grupo de Apoyo al Manejo de la endocarditis infecciosa en ESpaña* (GAMES). IE: infective endocarditis. CIED: cardiac implantable electronic device.

Of 5900 patients with possible or definite IE, 4531 had definite IE, 4064 had definite valvular IE, and 577 with MIE (14.2%) (Figure 1).

In patients with MIE, the most common locations were mitral (552, 95.7%) and aortic (550, 95.3%). MIE was mainly due to mitral-aortic involvement (507, 87.9%), right or left IE was only seen in 68 patients (11.8%), and tricuspid-pulmonary involvement was rare (2, 0.3%). MIE involved only native valves in 450 patients (78.0%). Table 1 shows the baseline characteristics and clinical events of our patients with valvular IE.

Compared with patients with SIE, patients with MIE had a similar age (69 vs. 67 years), were more frequently men (67% vs. 73%), and had a more common streptococcal etiology (29% vs. 33%).

**Figure 2:**
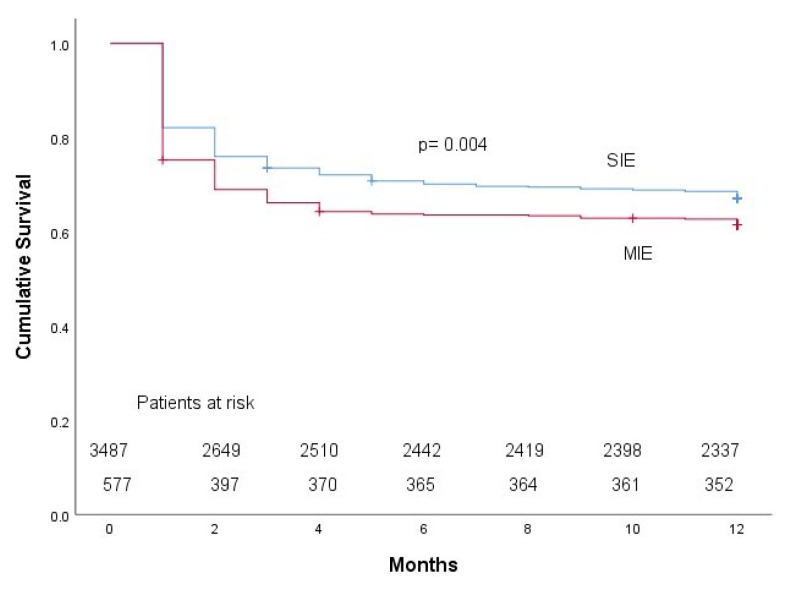
Kaplan–Meier curves for the cumulative survival of patients with valvular infective endocarditis (IE) according to the presence of single-valve IE (SIE) or multivalvular IE (MIE). *p*-value reflects the result of the log-rank test.

Complications were also more common in MIE, including intracardiac complications (36% vs. 50%) and heart failure (43% vs. 53%). MIE was associated with surgical indication (68% vs. 85%) and surgery (46% vs. 56%). Compared with patients with SIE, patients with MIE had higher in-hospital mortality (34% vs. 27%). MIE was an independent predictor of in-hospital mortality (odds ratio 1.3, 95% confidence interval 1.1–1.7) (Table 2A). One-year mortality was also higher in patients with MIE than in those with SIE (39% vs. 33%, Figure 2), although MIE did not have an independent association with one-year mortality (OR 1.1, 95% CI 0.9–1.4, *p* = 0.43) (Table 2B).

## 4. Discussion

In our large national cohort of valvular IE, one-seventh of patients had MIE, which was associated with a poor prognosis.

MIE can involve native and prosthetic valves. The primary event is bacterial adherence to damaged valves during bacteriemia, with later persistence and growth within the cardiac lesions causing local extensions and tissue damage [21]. MIE can be the result of a simultaneous infection in two previously damaged valves in a patient with persistent bacteriemia or, more often, sequential seeding of a previously damaged valve [11]. In other cases, the infection of the first valve creates a new valvular lesion. A jet of aortic regurgitation may damage and infect the anterior mitral leaflet, which can also cause mechanical complications in the leaflets and mitral valve apparatus [11,21,22]. Other proposed mechanisms are the formation of an anterior abscess spreading and the destruction of the mitral annulus, or the prolapsing aortic IE “kissing vegetation phenomenon” [23]. Bilateral IE is uncommon (12% in our series) and might be related to shunts produced by congenital heart diseases [3], intracardiac devices, or repeated injections by drug users.

The most common etiologies in our and other cohorts were streptococcal, staphylococcal, and enterococcal [2,3,4,5,6,7,8,9,11,24]. *S. viridans* infections more frequently occurred in MIE compared with SIE, a fact that has been previously reported [3,4,6,11,24]. However, the incidence of *Staphylococci* IE is increasing [25]. *S. gallolyticus* (*S. bovis*) also has a high frequency of multivalvular locations, especially in French registries [11], more often infecting advanced age patients with known heart disease.

Surgical treatment is frequently needed in IE and even more so in MIE. About 85% of our MIE patients had a surgical indication, a higher proportion than in SIE, although only in 56% of MIE patients was surgery finally performed. MIE patients develop heart failure more often than SIE patients, and heart failure is a common surgical indication [1]. In addition, patients with MIE frequently present extensive tissue destruction [24]. These two factors are probably related to the higher rate of surgical treatment in MIE than in SIE [3,4,6,9].

The prognostic influence of MIE is unclear. An association with poor outcomes has been described in some studies [3,4,5,12,13,14,15,26] but not in others [2,6,7,8,9,11,24]. Prosthetic IE is an important compounding factor when we compare MIE with SIE, as prosthetic IE is associated with a poor prognosis [4]. Table 3 summarizes the main information previously published regarding MIE. The mean age was higher in our cohort than in the published studies, probably because most previous data came from surgical series, and patients eligible for surgery are usually younger.

Comparisons of SIE and information regarding medically treated patients are scarce. Moreover, a surgical series of selected patients shows excellent results that do not reflect everyday clinical practice. Our series of MIE is the largest reported to date and includes both patients treated with and without surgery.

The limitations of this study should be noted. Local medical teams were responsible for IE management, including deciding on surgery, and any judgments may have been influenced by factors not registered in this study. In any case, our data come from a large national database and show a clear association of MIE with IE prognosis.

## 5. Conclusions

About one-seventh of the valvular IE patients had MIE, mainly due to mitral-aortic involvement. MIE is associated with a poor in-hospital prognosis. An early diagnosis and treatment of IE might avoid its spread to a second valve.

## Figures and Tables

**Table 1:** Baseline characteristics and clinical events in patients with valvular infective endocarditis (IE) according to the presence of single-valve IE (SIE) or multivalvular IE (MIE).

	SIE (3487)	MIE (577)	*p*
Age, mean (IQR ^1^)	69 (57–77)	67 (57–76)	0.273
Sex (Men)	2341 (67.1%)	421 (72.9%)	0.005
Location			
Aortic	1827 (52.4%)	550 (95.3%)	<0.001
Mitral	1428 (41.0%)	552 (95.7%)	<0.001
Tricuspid	177 (5.1%)	57 (9.9%)	<0.001
Pulmonary	50 (1.4%)	18 (3.1%)	0.003
Native IE	2326 (66.7%)	450 (78.0%)	<0.001
Prosthetic IE	1169 (33.5%)	194 (33.6%)	0.963
Etiology			
*Staphylococcus aureus*	842 (24.1%)	113 (19.6%)	0.017
Coagulase-negative staphylococci	618 (17.7%)	94 (16.3%)	0.402
*Enterococcus*	541 (15.5%)	109 (18.9%)	0.040
*Streptococcus*	999 (28.6%)	192 (33.3%)	0.024
*Candida*	50 (1.4%)	7 (1.2%)	0.676
Clinical course			
Vegetation present	2759 (79.1%)	493 (85.4%)	<0.001
Intracardiac complications	1261 (36.2%)	291 (50.4%)	<0.001
Perforation or rupture	533 (15.2%)	150 (25.9%)	<0.001
Pseudoaneurysm	242 (6.9%)	56 (9.7%)	0.018
Abscess	682 (19.5%)	151 (26.1%)	0.001
Intracardiac fistula	98 (2.8%)	26 (4.5%)	0.028
Vascular phenomenon	378 (10.8%)	60 (10.4%)	0.751
New heart murmur	1261 (36.2%)	263 (45.6%)	<0.001
Heart failure	1489 (42.7%)	305 (52.9%)	<0.001
Persistent bacteriemia	404 (11.6%)	69 (12.0%)	0.796
Central nervous system involvement	777 (22.3%)	140 (24.3%)	0.292
Embolization	834 (23.9%)	153 (26.5%)	0.177
Renal failure	1278 (36.7%)	220 (38.1%)	0.495
Septic shock	465 (13.3%)	94 (16.3%)	0.056
Sepsis	648 (18.6%)	105 (18.2%)	0.825
Indication for surgery	2360 (67.7%)	491 (85.1%)	<0.001
Cardiac surgery	1616 (46.3%)	325 (56.3%)	<0.001
Surgery indicated not performed	772 (22.1%)	168 (29.1%)	<0.001
Mean hospital stay (IQR)	36 (22–52)	38 (22–54)	0.368
Antibiotic treatment days, mean (IQR)	40 (28–46)	38 (21–45)	0.368
In-hospital mortality	937 (26.9%)	198 (34.3%)	<0.001
1-year mortality	1146 (32.9%)	222 (38.5%)	0.008
IE Recurrence in those alive	42 (1.6%)	7 (1.8%)	0.777

^1^ IQR: interquartile range.

**Table 2:** A. Independent predictors of in-hospital mortality in patients with valvular infective endocarditis (IE). B. Independent predictors of 1-year mortality in patients with valvular infective endocarditis (IE).

**(A)**		
	**OR (95% CI)**	***p***
Male sex	0.8 (0.7–0.9)	0.041
Charlson comorbidity index	1.12 (1.09–1.16)	<0.001
Heart failure	2.9 (2.5–3.4)	<0.001
Multivalvular IE	1.3 (1.1–1.7)	0.004
Severe sepsis	2.1 (1.8–2.6)	<0.001
*S. aureus*	1.7 (1.4–2.1)	<0.001
Nosocomial IE	1.6 (1.3–1.9)	<0.001
Intracardiac abscess	1.3 (1.1–1.7)	0.004
Age (years)	1.015 (1.008–1.022)	<0.001
**(B)**		
	**OR (95% CI)**	***p***
Mitral location	1.2 (1.0–1.4)	0.017
Charlson comorbidity index	1.15 (1.12–1.19)	<0.001
Heart failure	2.5 (2.2–2.9)	<0.001
Persistent bacteriemia	1.2 (1.0–1.5)	0.043
Severe sepsis	2.0 (1.7–2.4)	<0.001
*S. aureus*	1.4 (1.2–1.7)	<0.001
Nosocomial IE	1.6 (1.3–1.9)	<0.001
Intracardiac abscess	1.4 (1.1–1.7)	<0.001
Age (years)	1.015 (1.015–1.009)	<0.001

**Table 3:** Previous data and our cohort results in patients with multivalvular infective endocarditis (MIE).

First Author, Year	N and Cohort Type	MIE	Mean/Median Age (Years)	Main Etiologies	MIE in-Hospital Mortality
*Kim et al., 2000* [2]	77	14 (31%)	65	*S. aureus* 43% *S. viridans* 36%	21% overall 29% surgery
*Mihalhevic et al., 2001* [4]	63 MIE surgery	All	49	*S. viridans* 28% *S. aureus*	16%
*Gillinov et al., 2001* [6]	54 MIE surgery	All	50	*Streptococci* 70% *Staphylococci* 18%	0%
*David et al., 2007* [7]	383 IE surgery	101 (26%)	51	*S. aureus* 23% *S. viridans* 18%	12%
*Yao et al., 2009* [3]	388 IE surgery	48 (12%)	42	*S. viridans* 29% *S. aureus* 19%	13%
*Sheikh et al., 2009* [8]	90 MIE surgery	All	53	*S. aureus* 16% *S. viridans* 14%	15%
*Selton et al., 2010* [11]	300 IE 511 IE	42 (14%) 88 (17%)	58 60	*Streptococci* 64% *S. aureus* 14%	26% 23%
*López et al., 2011* [15]	680 IE	115 (17%)	58	*S. aureus* 22% Coagulase-negative *staphylococci* 18%	30%
*Ota et al., 2011* [9]	152 native valve IE surgery	35 (23%)	47	*S. aureus* 22% *S. viridans* 22%	9%
*Kim et al., 2013* [24]	90 native valve IE surgery	23 (25%)	47	*S. viridans* 70%	0%
*Our cohort*	4064 IE	577 (14%)	67	*S. viridans* 33% *S. aureus* 20%	34%

## Data Availability

Data available under request from SEICAV.

## Appendix A

**Members of GAMES: Hospital Costa del Sol**, (Marbella): Fernando Fernández Sánchez, José Mª García de Lomas, Gabriel Rosas, and Javier de la Torre Lima; **Hospital Universitario de Cruces**, (Bilbao): Elena Bereciartua, María José Blanco Vidal, Roberto Blanco, María Victoria Boado, Marta Campaña Lázaro, Alejandro Crespo, Laura Guio Carrión, Mikel Del Álamo Martínez de Lagos, Gorane Euba Ugarte, Ane Josune Goikoetxea, Marta Ibarrola Hierro, José Ramón Iruretagoyena, Josu Irurzun Zuazabal, Leire López-Soria, Miguel Montejo, Javier Nieto, David Rodrigo, Regino Rodríguez, Yolanda Vitoria, and Roberto Voces; **Hospital Universitario Virgen de la Victoria**, (Málaga): Mª Victoria García López, Radka Ivanova Georgieva, Guillermo Ojeda, Isabel Rodríguez Bailón, and Josefa Ruiz Morales; **Hospital Universitario Donostia-Poliklínica Gipuzkoa-IIS Biodonostia**, (San Sebastián): Ignacio Álvarez Rodríguez, Harkaitz Azkune Galparsoro, Elisa Berritu Boronat, Mª Jesús Bustinduy Odriozola, Cristina del Bosque Martín, Tomás Echeverría, Alberto Eizaguirre Yarza, Ana Fuentes, Miguel Ángel Goenaga, Muskilda Goyeneche del Río, Ángela Granda Bauza, José Antonio Iribarren, Xabier Kortajarena Urkola, José Ignacio Pérez-Moreiras López, Ainhoa Rengel Jiménez, Karlos Reviejo, Alberto Sáez Berbejillo, Elou Sánchez Haza, Rosa Sebastián Alda, Itziar Solla Ruiz, Irati Unamuno Ugartemendia, Diego Vicente Anza, Iñaki Villanueva Benito, and Mar Zabalo Arrieta; **Hospital General Universitario de Alicante**, (Alicante): Rafael Carrasco, Vicente Climent, Patricio Llamas, Esperanza Merino, Joaquín Plazas, and Sergio Reus; **Complejo Hospitalario Universitario A Coruña**, (A Coruña): Nemesio Álvarez, José María Bravo-Ferrer, Laura Castelo, José Cuenca, Pedro Llinares, Enrique Miguez Rey, María Rodríguez Mayo, Efrén Sánchez, and Dolores Sousa Regueiro; **Complejo Hospitalario Universitario de Huelva**, (Huelva): Francisco Javier Martínez; **Hospital Universitario de Canarias**, (Canarias): Mª del Mar Alonso, Beatriz Castro, Teresa Delgado Melian, Javier Fernández Sarabia, Dácil García Rosado, Julia González González, Juan Lacalzada, Lissete Lorenzo de la Peña, Alina Pérez Ramírez, Pablo Prada Arrondo, and Fermín Rodríguez Moreno; **Hospital Regional Universitario de Málaga**, (Málaga): Antonio Plata Ciezar, José Mª Reguera Iglesias; **Hospital Universitario Central Asturias**, (Oviedo): Víctor Asensi Álvarez, Carlos Costas, Jesús de la Hera, Jonnathan Fernández Suárez, Lisardo Iglesias Fraile, Víctor León Arguero, José López Menéndez, Pilar Mencia Bajo, Carlos Morales, Alfonso Moreno Torrico, Carmen Palomo, Begoña Paya Martínez, Ángeles Rodríguez Esteban, Raquel Rodríguez García, and Mauricio Telenti Asensio; **Hospital Clínic-IDIBAPS, Universidad de Barcelona**, (Barcelona): Manuel Almela, Juan Ambrosioni, Manuel Azqueta, Mercè Brunet, Marta Bodro, Ramón Cartañá, Guillermo Cuervo, Carlos Falces, Guillermina Fita, David Fuster, Cristina García de la Mària, Delia García-Pares, Marta Hernández-Meneses, Jaume Llopis Pérez, Francesc Marco, José M. Miró, Asunción Moreno, David Nicolás, Salvador Ninot, Eduardo Quintana, Carlos Paré, Daniel Pereda, Juan M. Pericás, José L. Pomar, José Ramírez, Irene Rovira, Elena Sandoval, Marta Sitges, Dolors Soy, Adrián Téllez, José M. Tolosana, Bárbara Vidal, and Jordi Vila; **Hospital General Universitario Gregorio Marañón**, (Madrid): Iván Adán, David Alonso, Juan Carlos Alonso, Ana Álvarez-Uría, Javier Bermejo, Emilio Bouza, Gregorio Cuerpo Caballero, Antonia Delgado Montero, Agustín Estévez, Ramón Fortuny Ribas, Esther Gargallo, Ana González Mansilla, Mª Eugenia García Leoni, Francisco Javier González Moraga, Víctor González Ramallo, Martha Kestler Hernández, Amaia Mari Hualde, Marina Machado, Mercedes Marín, Manuel Martínez-Sellés, Rosa Melero, Patricia Muñoz, Diego Monzón, María Olmedo, Álvaro Pedraz, Blanca Pinilla, Ángel Pinto, Cristina Rincón, Hugo Rodríguez-Abella, Marta Rodríguez-Créixems, Eduardo Sánchez-Pérez, Antonio Segado, Neera Toledo, Maricela Valerio, Pilar Vázquez, Eduardo Verde Moreno, and Sofía de la Villa; **Hospital Universitario La Paz**, (Madrid): Isabel Antorrena, Belén Loeches, Mar Moreno, Ulises Ramírez, Verónica Rial Bastón, María Romero, and Sandra Rosillo; **Hospital Universitario Marqués de Valdecilla**, (Santander): Jesús Agüero Balbín, Cristina Amado, Carlos Armiñanzas Castillo, Francisco Arnaiz de las Revillas, Manuel Cobo Belaustegui, María Carmen Fariñas, Concepción Fariñas-Álvarez, Marta Fernández Sampedro, Iván García, Claudia González Rico, Laura Gutierrez-Fernandez, Manuel Gutiérrez-Cuadra, José Gutiérrez Díez, Marcos Pajarón, José Antonio Parra, Ramón Teira, and Jesús Zarauza; **Hospital Universitario Puerta de Hierro**, (Madrid): Jorge Calderón Parra, Marta Cobo, Fernando Domínguez, Pablo García Pavía, Ana Fernández Cruz, Antonio Ramos-Martínez, and Isabel Sánchez Romero; **Hospital Universitario Ramón y Cajal**, (Madrid): Tomasa Centella, José Manuel Hermida, José Luis Moya, Pilar Martín-Dávila, Enrique Navas, Enrique Oliva, Alejandro del Río, Jorge Rodríguez-Roda Stuart, and Soledad Ruiz; **Hospital Universitario Virgen de las Nieves**, (Granada): Carmen Hidalgo Tenorio; **Hospital Universitario Virgen Macarena**, (Sevilla): Manuel Almendro Delia, Omar Araji, José Miguel Barquero, Román Calvo Jambrina, Marina de Cueto, Juan Gálvez Acebal, Irene Méndez, Isabel Morales, and Luis Eduardo López-Cortés; **Hospital Universitario Virgen del Rocío**, (Sevilla): Arístides de Alarcón, Encarnación Gutiérrez-Carretero, José Antonio Lepe, José López-Haldón, Rafael Luque-Márquez, Guillermo Marín, Antonio Ortiz-Carrellán, and Eladio Sánchez-Domínguez; **Hospital San Pedro**, (Logroño): Luis Javier Alonso, Pedro Azcárate, José Manuel Azcona Gutiérrez, José Ramón Blanco, Antonio Cabrera Villegas, Lara García-Álvarez, Concepción García García, and José Antonio Oteo; **Hospital de la Santa Creu i Sant Pau**, (Barcelona): Natividad de Benito, Mercé Gurguí, Cristina Pacho, Roser Pericas, and Guillem Pons; **Complejo Hospitalario Universitario de Santiago de Compostela**, (A Coruña): M. Álvarez, A. L. Fernández, Amparo Martínez, A. Prieto, Benito Regueiro, E. Tijeira, and Marino Vega; **Hospital Universitario Araba**, (Vitoria): Amaia Aguirre Quiñonero, Ángela Alonso Miñambres, Juan Carlos Gainzarain Arana, Sara González de Alaiza Ortega, Miguel Ángel Morán Rodríguez, Anai Moreno Rodríguez, Zuriñe Ortiz de Zárate, José Joaquín Portu Zapirain, Ester Sáez de Adana Arroniz, and Daisy Carolina Sorto Sánchez; **Hospital SAS Línea de la Concepción**, (Cádiz): Sánchez-Porto Antonio and Úbeda Iglesias Alejandro; **Hospital Clínico Universitario Virgen de la Arrixaca** (Murcia): José Mª Arribas Leal, Elisa García Vázquez, Alicia Hernández Torres, Ana Blázquez, Gonzalo de la Morena Valenzuela; **Hospital de Txagorritxu**, (Vitoria): Ángel Alonso, Javier Aramburu, Felicitas Elena Calvo, Anai Moreno Rodríguez, and Paola Tarabini-Castellani; **Hospital Virgen de la Salud,** (Toledo): Eva Heredero Gálvez, Carolina Maicas Bellido, José Largo Pau, Mª Antonia Sepúlveda, Pilar Toledano Sierra, and Sadaf Zafar Iqbal-Mirza; **Hospital Rafael Méndez,** (Lorca-Murcia):, Eva Cascales Alcolea, Ivan Keituqwa Yañez, Julián Navarro Martínez, and Ana Peláez Ballesta; **Hospital Universitario San Cecilio** (Granada): Eduardo Moreno Escobar, Alejandro Peña Monje, Valme Sánchez Cabrera, and David Vinuesa García; **Hospital Son Llátzer** (Palma de Mallorca): María Arrizabalaga Asenjo, Carmen Cifuentes Luna, Juana Núñez Morcillo, Mª Cruz Pérez Seco, and Aroa Villoslada Gelabert; **Hospital Universitario Miguel Servet** (Zaragoza): Carmen Aured Guallar, Nuria Fernández Abad, Pilar García Mangas, Marta Matamala Adell, Mª Pilar Palacián Ruiz, and Juan Carlos Porres; **Hospital General Universitario Santa Lucía** (Cartagena): Begoña Alcaraz Vidal, Nazaret Cobos Trigueros, María Jesús Del Amor Espín, José Antonio Giner Caro, Roberto Jiménez Sánchez, Amaya Jimeno Almazán, Alejandro Ortín Freire, and Monserrat Viqueira González; **Hospital Universitario Son Espases** (Palma de Mallorca): Pere Pericás Ramis, Mª Ángels Ribas Blanco, Enrique Ruiz de Gopegui Bordes, and Laura Vidal Bonet; **Complejo Hospitalario Universitario de Albacete** (Albacete): Mª Carmen Bellón Munera, Elena Escribano Garaizabal, Antonia Tercero Martínez, and Juan Carlos Segura Luque; **Hospital Universitario Terrassa:** Cristina Badía, Lucía Boix Palop, Mariona Xercavins, and Sónia Ibars; **Hospital Universitario Dr. Negrín** (Gran Canaria): Xerach Bosch, Eloy Gómez Nebreda, Ibalia Horcajada Herrera, Irene Menduiña Gallego, and Imanol Pulido; **Complejo Hospitalario Universitario Insular Materno Infantil** (Las Palmas de Gran Canaria): Héctor Marrero Santiago, Isabel de Miguel Martínez, Elena Pisos Álamo, and Daniel San Román Sánchez; **Hospital Universitario 12 de Octubre** (Madrid): Jorge Boan Pérez, Eva Mª Aguilar Blanco, Mercedes Catalán González, María Angélica Corres Peiretti, Andrea Eixerés Esteve, Laura Domínguez Pérez, Santiago de Cossío Tejido, Francisco Galván Román, José Antonio García Robles, Francisco López Medrano, Mª Jesús López Gude, Mª Ángeles Orellana Miguel, Patrick Pilkington, Yolanda Revilla Ostalaza, Juan Ruiz Morales, Sebastián Ruiz Solís, Ana Sabín Collado, Marcos Sánchez Fernández, Javier Solera Rallo, and Jorge Solís Martín; **Hospital Universitari de Bellvitge (L’Hospitalet de Llobregat):** Francesc Escrihuela-Vidal, Jordi Carratalà, Inmaculada Grau, Carmen Ardanuy, Dámaris Berbel, José Carlos Sánchez Salado, Oriol Alegre, Alejandro Ruiz Majoral, Fabrizio Sbraga, Arnau Blasco, Laura Gracia Sánchez, and Iván Sánchez-Rodríguez. **Hospital Universitario Fundación Jiménez Díaz** (Madrid): Gonzalo Aldamiz, Beatriz Álvarez, Alfonso Cabello Úbeda, Ricardo Fernández Roblas, Rafael Hernández, Victoria Andrea Hortigüela Martín, Andrea Kallmeyer, Cristina Landaeta Kancev, Miguel Morante Ruiz, Miguel Ángel Navas Lobato, Iris Martínez Alemany, Ana María Pello, Laura Prieto, and Marta Tomás Mallebrera; **Hospital Basurto** (Bilbao): Mireia de la Peña Triguero, Ruth Esther Figueroa Cerón, and Lara Ruiz Gómez; **Hospital del Mar** (Barcelona): Mireia Ble, Juan Pablo Horcajada Gallego, Antonio José Ginel, Inmaculada López, Alexandra Mas, Antoni Mestres, Lluís Molina, Ramón Serrat, Núria Ribas, Francisca Sánchez, Ana Silverio, Marina Suárez, Luisa Sorlí, Lluís Recasens, Manuel Taurón.
